# Concomitant progressive supranuclear palsy and chronic traumatic encephalopathy in a boxer

**DOI:** 10.1186/2051-5960-2-24

**Published:** 2014-02-21

**Authors:** Helen Ling, Eleanna Kara, Tamas Revesz, Andrew J Lees, Gordon T Plant, Davide Martino, Henry Houlden, John Hardy, Janice L Holton

**Affiliations:** 1 Reta Lila Weston Institute of Neurological Studies, Queen Square Brain Bank for Neurological Disorders, UCL Institute of Neurology, University College London, London, UK; 2 Department of Molecular Neuroscience, UCL Institute of Neurology, University College London, 1 Wakefield Street, London WC1N 1PJ, United Kingdom; 3 Sara Koe PSP Research Centre, Institute of Neurology, University College London, London, UK; 4 National Hospital for Neurology and Neurosurgery, Queen Square, UCL Institute of Neurology, University College London, London, UK; 5 Queen Elizabeth Hospital, Woolwich, South London NHS Trust, Department of Neurology, King’s College Hospital NHS Foundation Trust, London, UK; 6 Centre for Neuroscience and Trauma, Blizard Institute, Queen Mary University of London, London, UK

**Keywords:** Boxer, Dementia pugilistica, Chronic traumatic encephalopathy, Progressive supranuclear palsy, Tauopathy

## Abstract

We report the case of a 75-year-old ex-professional boxer who developed diplopia and eye movement abnormalities in his 60’s followed by memory impairment, low mood and recurrent falls. Examination shortly before death revealed hypomimia, dysarthria, vertical supranuclear gaze palsy and impaired postural reflexes. Pathological examination demonstrated 4-repeat tau neuronal and glial lesions, including tufted astrocytes, consistent with a diagnosis of progressive supranuclear palsy. In addition, neurofibrillary tangles composed of mixed 3-repeat and 4-repeat tau and astrocytic tangles in a distribution highly suggestive of chronic traumatic encephalopathy were observed together with limbic TDP-43 pathology. Possible mechanisms for the co-occurrence of these two tau pathologies are discussed.

## Background

Chronic traumatic encephalopathy (CTE), previously known as punch-drunk syndrome or dementia pugilistica, is a neurodegenerative tauopathy caused by repetitive and cumulative head trauma. CTE was first described in boxers [1] but can occur in other contact sports, such as steeplechase racing [2] and American football, and has also been described in people who have been repeatedly battered [3]. Pathological findings include generalized cortical atrophy, cavum septum pellucidum, extensive neurofibrillary tangles (NFTs) and astrocytic tangles composed of mixed 3-repeat (3R) and 4-repeat (4R) tau isoforms in the frontal and temporal cortices, often patchy and irregular, with predilection for perivascular regions [4] and in the depths of cerebral sulci with NFTs in the limbic regions, diencephalon and brainstem [5] but, in contrast to Alzheimer’s disease (AD), there is relatively little amyloid-β deposition (Aβ) [4-7].

PSP is a distinctive clinicopathological entity with established neuropathological diagnostic criteria [8-11]. PSP presents classically with postural instability and falls, slurred speech, a vertical supranuclear gaze palsy and a dysexecutive syndrome [12], but there are a number of well delineated atypical presentations [13-15].

We present the case of a retired professional boxer, who had been followed up for several years prior to the onset of his first neurological symptoms in a neuro-ophthalmology clinic.

## Materials and methods

### Patient and pathological material

Brain donors at the Queen Square Brain Bank for Neurological Disorders (QSBB), including the present case, register for a brain donor program approved by a London Multi-Centre Research Ethics Committee and tissue is stored for research under a license from the Human Tissue Authority. A written and signed consent for the publication of this case report was obtained from the patient’s wife.

Tissue blocks were taken using standard protocols. Established pathological diagnostic criteria for CTE [5] and PSP [8-10,16] were used. Haematoxylin and eosin (H&E) were used to assess neuronal loss and gliosis in the basal ganglia and substantia nigra (SN). Bielschowsky’s silver staining was used to detect neurofibrillary tangle and neuritic plaque pathology. Immunohistochemistry with antibodies to phosphorylated tau (AT8), 3R tau and 4R tau ^16^, Aβ peptide, α-synuclein, p62 and TAR DNA-binding protein-43 (TDP-43) was performed using a standard avidin-biotin method. The neuropathological findings were reviewed by 2 neuropathologists (TR, JLH).

### Genetic analysis

Genomic DNA was extracted from frozen brain tissue of the index case and from the plasma sample of his older brother, who developed corticobasal syndrome, following informed consent. Sanger sequencing was performed using standard procedures as previously described [17] for *LRRK2* (exons 24, 25, 27, 29, 31, 35, 36, 41 and 48), *MAPT* (exons 1-13) and Progranulin (*GRN*) (exons 1-13). *MAPT* haplotypes were determined [18]. *APOƐ* genotype was determined [19]. Larger genomic rearrangements in *LRRK2* were assessed through the Multiplex Ligation-dependent Probe Amplification (MLPA) kit P052C (MRC Holland) [20]. *C9orf72* repeat expansion was analyzed using the reversed-prime PCR method [21].

## Case presentation

At age 64, the patient developed sudden onset horizontal double vision which was diagnosed as a right microvascular ischaemic sixth nerve palsy and his blood pressure was noted to be 190/110. Diabetes mellitus was excluded. Antihypertensive medications were prescribed and his diplopia almost completely resolved. In the following year, he had a further episode of sudden onset horizontal diplopia caused by a left lateral rectus paresis which persisted for five months with again a good recovery apart from occasional double vision on looking into the distance which improved with prism glasses. A diagnosis of recurrent microvascular ischaemic sixth nerve palsy was made. Ocular examination revealed esotropia for distance and exophoria for near vision. There was very mild underaction of the right lateral rectus muscle. In addition, slow vertical saccades were noted and very mild unsteadiness in tandem walking was observed. Magnetic resonance imaging (MRI) of the brain showed mild periventricular white matter changes compatible with small vessel disease. The unexpected finding of slow vertical saccades was considered as a possible sign of early PSP and the patient was then followed annually at Moorfields Eye Hospital.

At age 69, the patient began to fall backwards, developed hypophonia, mild swallowing difficulties with pooling of saliva and had progressive difficulty moving his eyes up and down. Examination showed poor convergence, slow vertical saccades and mild postural instability with retropulsion on pull test. In his early 70’s, he developed memory impairment. He also had increasing hearing difficulties and a neuro-otological review identified a significant right canal paresis on caloric testing indicative of a right peripheral vestibular dysfunction and pure tone audiometry confirmed bilateral moderate to severe gently sloping sensorineural hearing loss. He was referred for vestibular rehabilitation and prescribed hearing aids.

**Figure 1:**
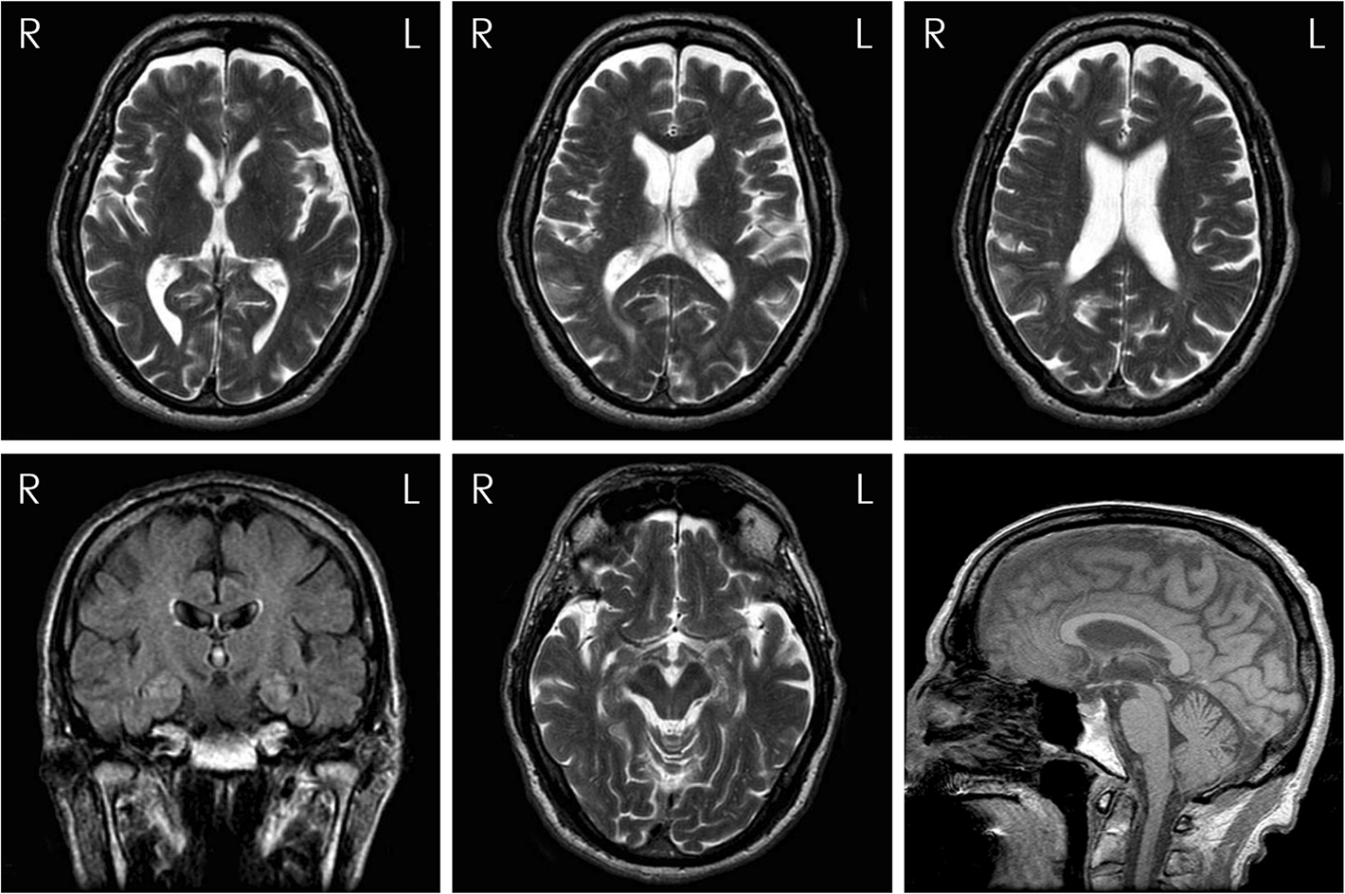
**Axial T2, coronal FLAIR and sagittal T1 brain magnetic resonance images when the patient was 72 years old.** Generalised volume loss with distinct midbrain tegmental atrophy is noted. There are scattered white matter foci of abnormal signal in keeping with small vessel disease.

**Figure 2:**
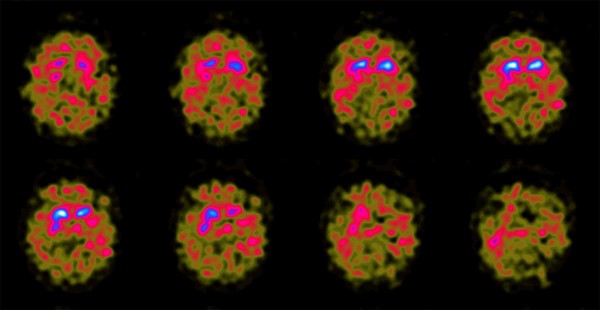
**Abnormal DAT-SPECT.** [123I]FP-CIT SPECT images showed bilateral and symmetrical reduced tracer uptake in the striatum indicative of nigrostriatal degeneration.

At age 73, he scored 25 out of 30 on MMSE; losing 1 point on recall, 3 points on attention span and 1 point on repetition. Full neuropsychological examination revealed executive inefficiency and cognitive slowness indicative of mild anterior and subcortical dysfunction. He had facial hypomimia with frontalis overactivity, dysarthria and postural instability. Eye movement examination revealed an esotropia for near and distance and slow hypometric vertical saccades especially on downgaze. Vertical eye movements were full on oculocephalic testing. Square wave jerks intruded on fixation and horizontal smooth pursuit was saccadic. His jaw jerk was brisk and tongue movements were slow and spastic. Haematological and biochemical blood tests and cerebrospinal fluid study, including Whipple’s PCR, were all normal. Genetic testing for spinocerebellar ataxia (SCA) 2, 3 and 6 and common mitochondrial (mtDNA) mutations was negative. Repeat MRI of the brain (Figure 1) showed generalized volume loss especially in the midbrain with distinct tegmental atrophy in addition to the scattered foci of abnormal signal in the white matter observed in the previous scan. Dopamine transporter scan, [123I]FP-CIT SPECT, showed bilateral reduced tracer uptake in the striatum (Figure 2).

The patient became progressively more withdrawn and was falling more frequently in the backward direction and required a frame to mobilise. At age 74, examination revealed absence of convergence and total loss of vertical eye movements. There were marked hypophonia, axial rigidity, mild retrocollis and presence of frontal release signs. He could no longer communicate, he developed severe dysphagia with aspiration and started losing weight and required help with all his daily activities. He died of right lobar pneumonia at age 75.

**Figure 3:**
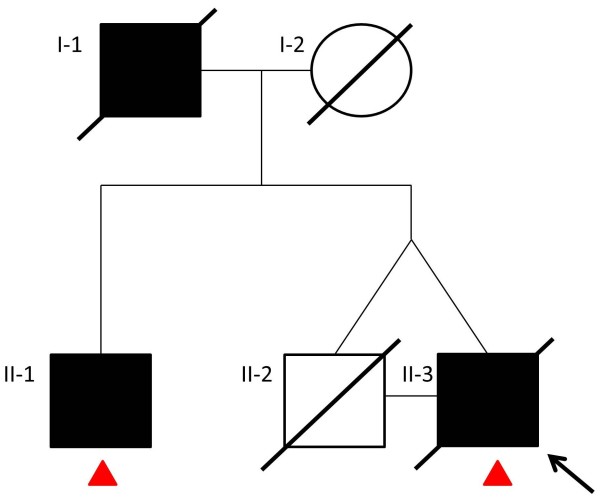
**Family tree.** The arrow indicates the index patient. Filled symbols represent affected family members. Red triangles indicate family members in whom sequencings of *MAPT*, Progranulin, *LRRK2* and *C9orf72* repeat expansion were negative and tau haplotype was H1/H1 and ApoE genotype was E3/E3.

Of relevance in the clinical history was that he had begun amateur boxing in his teenage years and he boxed professionally in the heavyweight category between the ages of 20 and 30. During his professional boxing career, he undertook 31 fights, including 16 wins, 1 draw, and 14 defeats, 13 of which were lost by knockout. After retiring from boxing, he worked as a boxing trainer for a number of years. At the age of 65, he was diagnosed with hypertension and hyperlipidaemia. He never smoked and only drank alcohol occasionally. His father, who also boxed as an amateur, died of a dementing illness aged 86 and his mother died of cerebrovascular disease aged 67. His identical twin brother also boxed professionally for 17 years and had 55 fights, including 40 wins, 1 draw, and 14 defeats, 8 of which were lost by knockout. The twin brother died at the age of 76 without ever having any neurological or psychiatric sequelae from boxing or any other neurological disease. His older brother, who has never boxed and is now aged 82, had a two-year history of progressive limb apraxia and apraxia of speech. He was reviewed by the author (HL) and the diagnosis of corticobasal syndrome was made (Figure 3).

### Neuropathological findings

The whole brain weight was 1311 grams. Mild dilatation of the frontal horn of the lateral ventricle and mild reduction in bulk of white matter of the frontal lobe were noted. The major macroscopic findings were mild reduction in bulk of the globus pallidus and subthalamic nucleus with pallor of the substantia nigra, reduction in height of the midbrain and pontine tegmenta and blurring of the dentate nucleus. The corpus callosum, thalamus, hypothalamus and mammillary body appeared normal. The septum pellucidum was torn due to post-mortem artefact but cavum septum pellucidum was not apparent.

**Figure 4:**
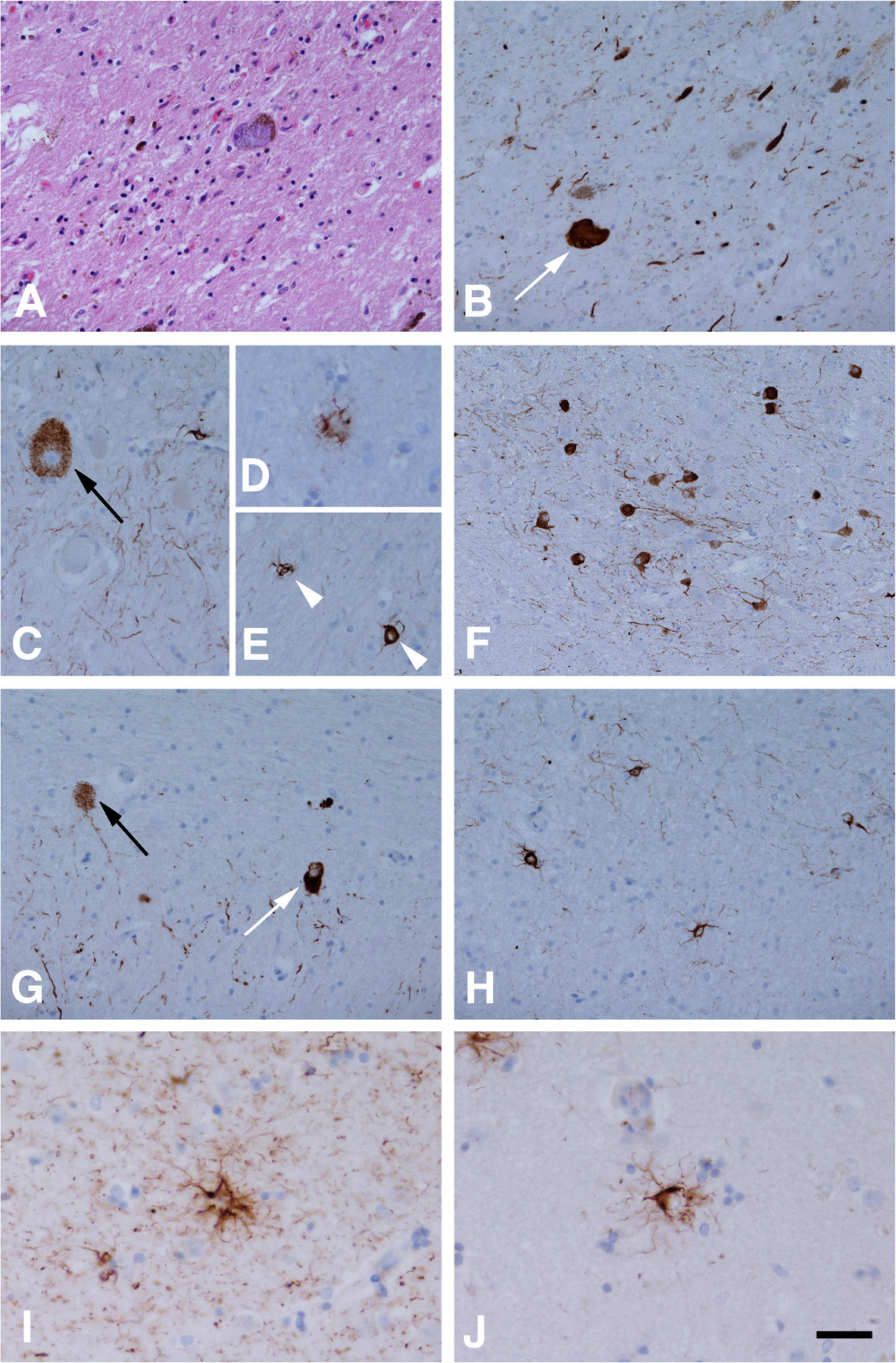
**Illustrations of the substantia nigra (SN) and progressive supranuclear palsy (PSP) pathology.** There is severe neuronal loss and gliosis **(A)** with neurofibrillary tangles (NFTs) (white arrow, **B**) in the SN. Tau immunohistochemistry demonstrates pre-tangles (PreT, black arrow, **C**) and occasional tufted astrocytes (TAs) in the motor cortex **(D)**, and coiled bodies (CBs, arrowheads, **E**) in the posterior frontal cortical white matter, NFTs and neuropil threads (NTs) in the pontine base **(F)** and PreTs (black arrow, **G**), NFTs (white arrow, **G**) and threads in the dentate nucleus **(G)**, CBs and fine NTs in the cerebellar white matter **(H)**, and, TAs in the caudate nucleus **(I, J)**. TAs in the motor cortex **(F)** and caudate **(J)** are stained positive using 4-repeat tau antibody but not 3-repeat tau antibody. **A**: H&E, **B**, **C**,-**E**, **G**-**I**: tau immunohistochemistry (AT8), **F & J**: 4-repeat tau immunohistochemistry. Bar in **J** = 50 μm in **A**-**C**, **E**, **G**, **H** = 25 μm in **D**, **I**, **J** and = 100 μm in **F**.

Histological examination showed severe neuronal loss in the substantia nigra (SN) pars compacta. There was also neuronal loss and gliosis in the subthalamic nucleus (STN), locus coeruleus, dentate nucleus and mild Purkinje cell depletion in the cerebellar cortex. Tau immunohistochemistry revealed NFTs, pre–tangles, coiled bodies and neuropil threads in the SN, STN, brainstem nuclei, pons, cerebellar white matter and neocortex. Tufted astrocytes were noted in the striatum and neocortex. Tau lesions in the basal ganglia and brainstem were composed of 4R tau isoforms. These features fulfilled the diagnostic criteria of PSP (Table 1, Figure 4) [8-10,16].

**Table 1:** Distribution of immunoreactive inclusions positive to AT8 and TDP antibodies

	**Tau-immunoreactive lesions**							**TDP-43-immunoreative lesions**		
	**Neuritic plaques**	**NFTs & PreTs**	**Coiled bodies**	**Neuropil threads**	**Tufted astrocytes**	**Perivascular astrocytic tangles**	**Subpial astrocytic tangles**	**NCIs**	**NIIs**	**Threads**
Anterior frontal	+	++	++	++	+	+	++	-	-	-
Parietal	+	+	++	++	+	+	+	-	-	-
Temporal cortex	+	+	+	++	+	-	++	-	-	-
Hippocampus	-	+++	+	+++	-	-	+	+	+	+
Amygdala	-	+++	-	+++	-	+	NA	-	-	+
Caudate	-	+	+	+++	++	+	NA	-	-	-
Putamen	-	+	+	+	+	-	NA	-	-	-
Globus pallidus	-	+	++	+	-	-	NA	-	-	-
Internal capsule	-	NA	++	+++	-	-	NA	-	-	-
STN	-	++	++	+++	-	-	NA			
Substantia nigra	-	++	++	+++	-	-	NA	-	-	-
Red nucleus	-	++	+++	+++	-	-	NA	-	-	-
Oculomotor nucleus	-	++	-	+++	-	-	NA	-	-	-
Pons-tegmentum	-	++	+++	+++	-	-	NA			
Pons-base	-	++	+	+++	-	-	-			
Locus ceruleus	-	+++	+	+++	-	-	NA			
Medullary tegmentum	-	++	+++	+++	-	-	+	+	-	+
Inferior olive	-	++	+	++	-	-	-			
Dentate nucleus	-	++	-	++	-	-	NA			
Cerebellar wm	-	++	NA	++	-	-	-			

(-) none; (+) sparse; (++) moderate; (+++) frequent, NA-not applicable; blank spaces indicate that immunohistochemistry staining was not performed in the corresponding region; 3R: 3-repeat tau isoform; 4R: 4-repeat tau isoform: Aβ: amyloid beta; AT8: phosphorylated tau; NCIs: neuronal cytoplasmic inclusions; NFTs: neurofibrillary tangles, NIIs: neuronal intranuclear inclusions; preTs: pretangles; STN: subthalamic nuclei; wm: white matter.

**Figure 5:**
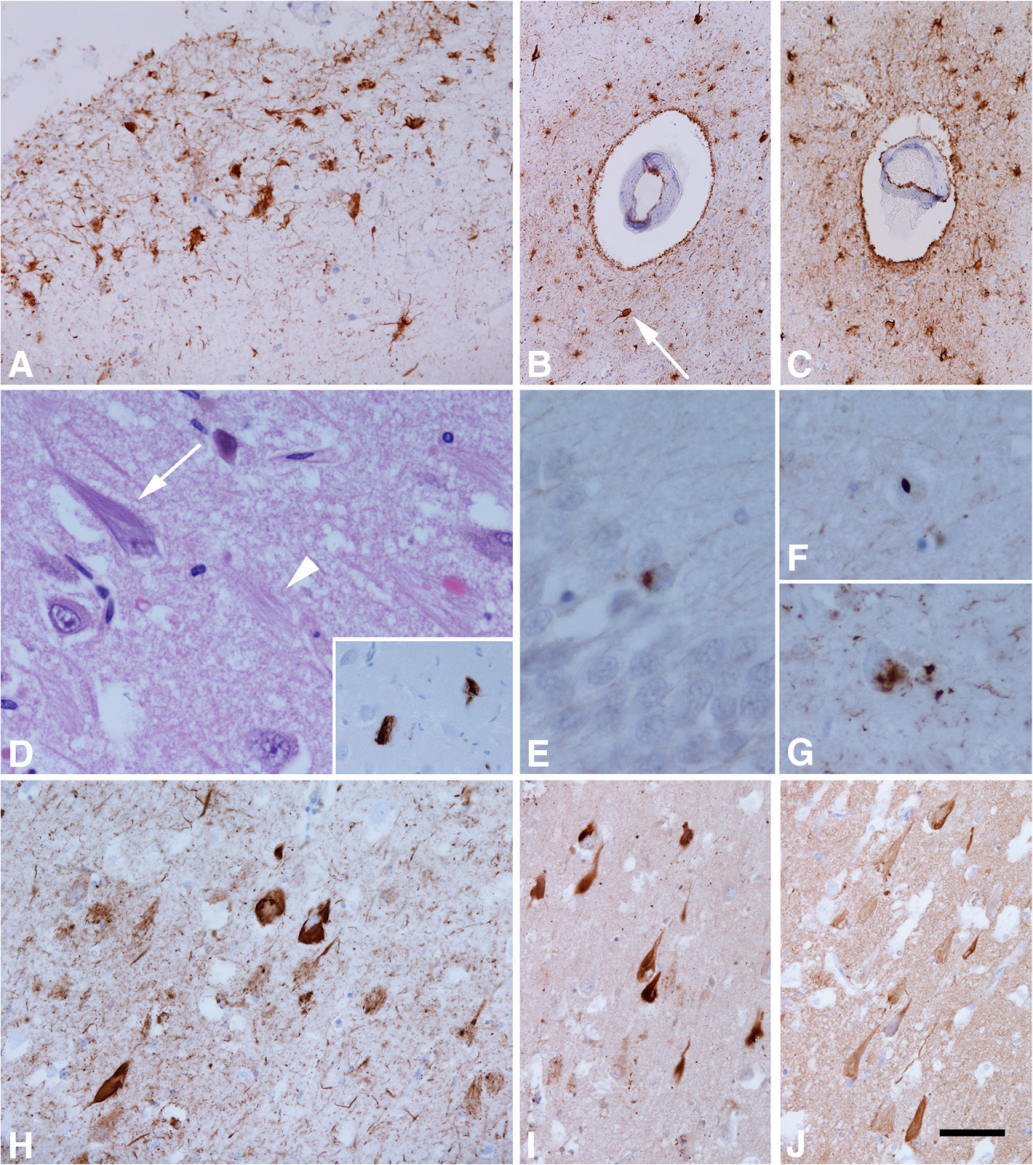
**Illustrations of chronic traumatic encephalopathy (CTE) pathology.** Tau immunohistochemistry demonstrates subpial astrocytic tangles in the depth of sulci of the frontal cortex **(A)**, perivascular astrocytic tangles in the insula **(B)** and parietal cortices **(C)** and neurofibrillary tangles (NFTs) are occasionally observed in the perivascular region (white arrow, **B**). There is marked neuronal loss with ghost tangles (arrowhead) and NFTs (arrow) in the CA1 hippocampal subregion on H&E **(D)** and amyloid-β deposition on the ghost tangles can be observed (inset in **D**). TDP-43 immunohistochemistry shows occasional neuronal cytoplasmic inclusions (NCIs) in the dentate fascia of the hippocampus **(E)**, a neuronal intranuclear inclusion (NII) in the entorhinal cortex **(F)**, and, occasional NCIs and threads in the CA1 hippocampal subregion **(G)**. Extensive tau-immunoreactive NFTs and neuropil threads (NTs) are observed in CA1 **(H)** and throughout the hippocampal formation and these NFTs and NTs are stained positive using either 3-repeat **(I)** and 4-repeat tau antibodies **(J)**. **A**, **B**, **C**, **H**: tau immunohistochemistry (AT8), **D**: H&E, Inset in **D**: Aβ immunohistochemistry, **E**-**G**: TDP-43 immunohistochemistry, **I**: 3-repeat, and **J**: 4-repeat tau immunohistochemistry. Bar in **J** = 50 μm in **A**, **H**-**J**, = 100 μm in **B** and = 25 μm in **D**-**G**.

In addition to these findings, there was marked loss of pyramidal neurons in the CA1 subregion of the hippocampus and subiculum accompanied by frequent NFTs and ghost tangles. Tau immunohistochemistry demonstrated that extensive NFTs and neuropil threads were present throughout the rest of the hippocampal formation, including CA2, CA3 and CA4 subregions, and extending into the fusiform gyrus. There were moderate numbers of pre-tangles and occasional NFTs in the dentate fascia. These NFTs contained 4R tau isoforms, whereas 3R tau immunohistochemistry was more restricted in distribution and was positive in NFTs in the CA1 subregion, subiculum, entorhinal and transentorhinal cortices. The amygdala was similarly affected by tau pathology. Ghost tangles, representing residual NFTs lying in the neuropil following neuronal death, were frequently observed in the CA1 subregion and subiculum visible in H&E stained sections and using Bielschowsky’s silver impregnation. These were also tau and Aβ immunoreactive, the latter indicating secondary Aβ deposition on extracellular protein aggregates. In neocortical regions foci of subpial tau-positive astrocytic tangles were observed, typically in the depths of sulci and occasionally with a perivascular distribution within the cortex. Moderate superficial NFTs in the temporal cortex were noted. These findings were consistent with coexisting chronic traumatic encephalopathy (CTE) pathology (Table 2, Figure 5) in additional to the PSP-tau pathology described above. The distribution of CTE pathology best corresponds to stage III according to McKee’s proposed criteria [5], although the NFTs in the hippocampus may partly be caused by AD-related pathology.

**Table 2:** Differentiating pathological and biochemical features between PSP and CTE

**Pathological features**	**PSP**	**CTE**
Tau protein isoform profile	-4-repeat predominant tau	-Both 4- and 3-repeat tau
		-4-repeat predominant astrocytic tangles in subpial and periventricular regions
Characteristic features	-Neuronal (NFTs, neuropil threads) and glial pathology (tufted astrocytes, coiled bodies) in a typical distribution	-Perivascular NFTs locate at depths of sulci and in superficial cortical layers
	-Neuronal loss in STN and dentate nucleus	-Subpial, perivascular and periventricular astrocytic tangles
		-Relatively mild Aβ pathology
		-Ghost tangles in limbic region and temporal neocortex
Shared features	-Associated TDP-43 related pathology limited to limbic region	-TDP-43 pathology in CTE tends to be more widespread involving cortical region in stage III and IV
	-Neuronal loss in SN and LC	-Neuronal loss in SN and LC
Distribution of hyperphosphorylated tau pathology	-SN, STN, GP, pons	-Cortical regions including frontal cortex, medial temporal lobe, thalamus and brainstem
	-Striatum, oculomotor complex, medulla, dentate nucleus, inferior olive and neocortex	

CTE: chronic traumatic encephalopathy, GP: globus pallidus, LC: locus ceruleus, NFT: neurofibrillary tangle, PSP: progressive supranuclear palsy, SN: substantia nigra, STN: subthalamic nucleus.

TDP-43-immunoreactive neuronal cytoplasmic inclusions (NCIs) and threads were identified in small numbers in the dentate fascia, entorhinal and transentorhinal cortices with sparse threads/neurites in the amygdala, and medullary tegmentum. TDP-43-positive neuronal intranuclear inclusions (NIIs) were rare in the entorhinal and transentorhinal cortices. There were no TDP-43 inclusions in the primary motor cortex and the XIIth cranial nerve nucleus. The spinal cord was not available for examination.

Aβ immunohistochemistry demonstrated parenchymal deposition in the neocortex where there were modereate numbers of diffuse deposits with sparse neuritic plaques and also in the hippocampus and striatum corresponding to Thal phase 3 [22]. The distribution of tau pathology could be considered to correspond to Braak and Braak stage III [23]. These changes corresponded to an ‘intermediate’ level of AD pathologic change (A2, B2, C1) according to the 2012 NIA-Alzheimer Association guidelines [24].

Mild small vessel disease was evidenced by the findings of hyaline mural thickening of the blood vessels in the subcortical white matter. There were no Lewy bodies, cerebral amyloid angiopathy, argyrophilic grains, or p62-positive ‘star-shaped’ inclusions in the hippocampus or small ‘dot-like’ structures in the cerebellar granule cells of the type associated with C9ORF72 repeat expansion.

In summary, the neuropathological diagnoses of this case were 1) PSP, 2) CTE stage III, 3) limbic TDP-43 proteinopathy, 4) intermediate level of AD pathologic change and 5) mild small vessel disease.

### Genetic findings

The *MAPT* haplotype of this patient was H1/H1 and *APOE* genotype was E3/E3. *LRRK2*, *MAPT*, progranulin sequencings and *C9orf72* repeat expansions were all negative. No large genomic rearrangements in *LRRK2* were detected. The genetic findings of the older brother who has developed corticobasal syndrome were similarly negative.

## Discussion

We report the case of a professional boxer who visited a neuro-ophthalmology clinic following the development of bilateral sequential lateral rectus palsy in his 60’s due to microvascular ischaemic insults. Neuro-ophthalmological review at the time led to detection of slow vertical saccades, which were considered to be the harbinger of an unrelated neurodegenerative disease. A few years later the patient experienced postural instability and backward falls along with progressive onset of other clinical features highly suggestive of Richardson’s syndrome (RS), the classical presentation of PSP [14]. The neuroimaging findings of midbrain atrophy and reduced striatal tracer uptake on dopamine transporter scan were also supportive of the clinical diagnosis of PSP [25]. Post-mortem examination confirmed the neuropathological findings of both PSP and CTE. The concomitant CTE-tau pathology in the hippocampus may have contributed to the cognitive decline. Clinical presentations of CTE in older individuals may be indistinguishable from AD with episodic memory impairment and executive dysfunction being more common than behavioural or mood changes [26].

Asymptomatic CTE can be observed in 11% of all CTE cases and is associated with early CTE pathology [5]. Moreover, there have been no pathologically confirmed CTE cases that present with a PSP-RS phenotype reported in the literature. Ocular abnormalities can be observed in CTE but their characteristics are not well defined [27]. The cognitive and behavioural symptoms and motor impairments in CTE can sometimes mimic other neurodegenerative disorders such as AD, frontotemporal dementia or Parkinson’s disease. Nevertheless, CTE tends to progress relatively slowly with a disease course spanning over three to four decades [5,27-29]. Cognitive impairments in our patient were consistent with a PSP-like dysexecutive syndrome. The patient never experienced any behavioral changes or neuropsychiatric symptoms that would have suggested early presentation of CTE prior to the onset of his diplopia. Cavum septum pellucidum or septal fenestrations, markers of head trauma, were absent in this case [29].

Repetitive head injury is the only known definitive risk factor for CTE but not all individuals who were exposed to repetitive head injury develop CTE [27]. A prospective study found that 68 out of 85 (80%) participants with histories of repetitive head injury had CTE pathology [5]. Apo E4 allele, which increases the risk of developing AD, has been linked with poor long-term neurological outcome after severe traumatic brain injury [30] and more severe CTE-related neurological deficits in boxers [31]. However, more studies are required to confirm Apo E4 as a susceptibility allele for CTE. Notably, this patient carried Apo E3/E3, the most common genotype in the population, may have had a protective effect from severe CTE-related neurological deficits [32]. Other putative risk factors for CTE, which were also observed in our patient, including family history of dementia, professional rather than amateur boxing, head trauma during youth and prolonged exposure will require further verification [27].

Head trauma is a known risk factor for certain neurodegenerative conditions such as AD [33], amyotrophic lateral sclerosis (ALS) [34] and Parkinson’s disease [35], but has never been firmly linked with PSP [36]. It is possible that our patient had subclinical CTE and subsequently developed another tauopathy, PSP, in later life by chance. However, a recent large clinicopathological series revealed that over a third of CTE cases had co-morbid neurodegenerative diseases; of the 68 CTE cases studied, 11 had Lewy body diseases, 8 had motor neuron disease, 7 had AD, 2 had FTLD-TDP, 1 had Pick’s disease and 1 had PSP [5]. The latter case had stage II CTE and concomitant PSP pathologies and died in his 70’s [5], but the clinical features were not elaborated. Another prevalence study identified one case of PSP among 704 retired Thai boxers [37]. It is plausible that either the CTE-tau pathology triggers the molecular pathways resulting in the accumulation of PSP-tau pathology in later life or both CTE and PSP share common risk factors, notably head trauma and axonal injury [28,38]. Further studies are required to establish any causative relationship between mild head injury and PSP.

The family history of this patient was intriguing. His identical twin brother, who would have also carried the potentially protective Apo E3/E3 alleles, had a long professional boxing career but never developed any neurological or psychological sequelae. The patient’s older brother had a progressive neurodegenerative disorder compatible with corticobasal syndrome. The underlying pathologies of corticobasal syndrome are heterogeneous, the most common of which are PSP, AD, FTLD and corticobasal degeneration (CBD) [39]. In both PSP and CBD, the possession of H1 allele of the *MAPT* gene and, in particular, the H1/H1 genotype is a risk factor which was also detected in both the patient and his older brother [40,41]. Although PSP and CBD are sporadic conditions, there have been reports of familial aggregation of parkinsonism in PSP [42] and a recent genome-wide association study (GWAS) identified variants within several loci increasing the risk for PSP [43]. Interestingly, Apo E4 allele frequency is lower in PSP than in controls [43,44]. All other gene sequencings, including *LRRK2*, *MAPT*, progranulin and *C9orf72* repeat expansions, were negative in the patient and his older brother, indicating there was no known, common/conventional monogenetic cause to their neurological conditions. There have been two previous reports on familial PSP/CBD with histopathological confirmation; *MAPT* sequencing was negative in the affected family members in both families and, in the more recent series, progranulin sequencing was also negative [45,46]. Future genetic analysis on CTE cases with co-existing neurodegenerative conditions and exome sequencings on the familial CBD/PSP cases are warranted.

TDP-43 is a major disease protein in FTLD and ALS and is occasionally observed in Lewy body disorders and tauopathies such as CTE and PSP [6,47-50]. TDP-43 pathology tends to localise in the limbic system when it co-exists in other neurodegenerative disorders; with an exception of advanced stages of CTE (stages III and IV), where TDP-43 pathology may be widespread involving the cortices, medial temporal lobe and brainstem in most cases [6]. In a subset of CTE cases, TDP-43 positive inclusions and neurites are found in the anterior horns of the spinal cord and motor cortex in association with corticospinal tract degeneration and loss of anterior horn cells, giving an ALS-like clinical picture [7]. TDP-43 inclusions in our case were sparse and confined to the limbic system [50]. TDP-43 immunoreactivity may modify clinical features in AD and other types of dementia [51] and is closely associated with hippocampal sclerosis [51,52]. The cause and mechanism of TDP-43 inclusions in tauopathies remain elusive but it is postulated that tau aggregates may promote aggregation of TDP-43 pathology through cross-seeding [28,38]. There is also evidence to show that the accumulation of TDP-43 in CTE brains may be part of a physiological injury response to traumatic brain injury [53].

Following the description of the ‘punch drunk’ symptom spectrum in boxers by Martland in 1928 [1] and motor deficits and mental confusion, termed dementia pugilistica, by Millspaugh in 1937 [54], Corsellis et al in 1973 published the neuropathological findings of 15 boxers and concluded that dementia pugilistica was a neuropathologically distinct disorder despite its similarities to AD [29]. The NFTs in CTE and AD are biochemically the same, containing both 3R and 4R tau [55]. However, tau pathologies in CTE tend to cluster around blood vessels, locate in the depths of the sulci and in the superficial cortical layers, are patchy and irregularly distributed and Aβ deposits are relatively scanty [4,56,57]. These pathological features were used in our case to distinguish CTE pathology from Alzheimer-related tau pathology, although overlaps can occur in the hippocampus and temporal neocortex. The clustering of astrocytic tangles in the subpial and perivascular regions in CTE, as observed in this case, is topographically distinct from thorny astrocytes in the medial temporal lobe reported in ageing and AD (Figure 5) [27]. In CTE stages III and IV, the loss of pigmented neurons in SN and locus coeruleus is common, which can overlap with PSP pathology, however the STN tends to be preserved in CTE but not in PSP [5,9]. In slowly progressive tauopathies such as CTE and post-encephalitic parkinsonism, ghost tangles are formed as a result of neuronal death and the formation of extracellular NFTs and can be observed in the limbic system and the temporal neocortex with evidence of Aβ deposition as in our case (Figure 5) [58,59].

## Conclusion

We illustrate the case of a former boxer who had been a clinical and pathological diagnostic challenge. His clinical and post-mortem diagnoses were consistent with classical PSP. However, his bilateral sequential lateral rectus palsy, peripheral vestibular dysfunction and long history of repetitive head injury during his boxing career were red herrings that had complicated the clinical picture. Pathologically, it had also been a challenge to dissect the concomitant PSP, CTE and AD-related tau pathologies.

## Abbreviations

Aβ: Amyloid beta; AD: Alzheimer’s disease; ALS: Amyotrophic lateral sclerosis; CB: Coiled body; CERAD: Consortium to Establish a Registry for Alzheimer’s disease; CTE: Chronic traumatic encephalopathy; DaTSCAN: [123I]FP-CIT SPECT images; FTLD: Frontotemporal lobar degeneration; GP: Globus pallidus; H&E: Haematoxylin and eosin; MMSE: Mini-mental state examination; MRI: Magnetic resonance imaging; NFT: Neurofibrillary tangle; NP: Neuritic plaque; PSP: Progressive supranuclear palsy; QSBB: Queen Square Brain Bank for Neurological Disorders; RS: Richardson’s syndrome; SCA: Spinocerebellar ataxia; SN: Substantia nigra; STN: Subthalamic nucleus; TA: Tufted astrocyte; TDP-43: Transactive response DNA binding protein with a molecular weight of 43-kDa; VOR: Vestibulo-ocular reflex; 3R: 3-repeat; 4R: 4-repeat.

## Competing interests

All authors declare that they have no competing interests.

## Authors’ contributions

HL reviewed the case notes, examined the patient’s brother, reviewed the neuropathological findings and drafted the manuscript. EK carried out genetic study and drafted the manuscript. TR reviewed the neuropathological findings and provided critique of the manuscript. AL reviewed the patient’s clinical information and provided critique of the manuscript. GP looked after the patient since his first presentation and provided critique of the manuscript. DM looked after the patient’s brother and provided critique of the manuscript. HH advised on genetic studies and provided critique of the manuscript. JH advised on genetic studies and provided critique of the manuscript. JH reviewed the neuropathological findings and provided critique of the manuscript. All authors read and approved the final manuscript.
